# *Ric-8A* gene deletion or phorbol ester suppresses tumorigenesis in a mouse model of *GNAQ*^Q209L^-driven melanoma

**DOI:** 10.1038/oncsis.2016.45

**Published:** 2016-06-27

**Authors:** B R Patel, G G Tall

**Affiliations:** 1 Department of Pharmacology, University of Michigan Medical Center, Ann Arbor, MI, USA

## Abstract

The heterotrimeric G protein α subunit oncogenes *GNAQ* or *GNA11* carry Q209X or R183X activating mutations and are present with ~90% frequency in human uveal melanomas. Forced expression of *GNAQ/11*^Q209L^ in melanocytes is sufficient to drive metastatic melanoma in immune-compromised mice. No known drugs directly target these oncogenic G proteins. Ric-8A is the molecular chaperone that selectively folds Gαq/i/13 subunits. Targeting Ric-8A serves as a rational, yet unexplored approach to reduce the functional abundance of oncogenic Gαq/11 in order to blunt cancer signaling. Here, using mouse melanocyte cell graft tumorigenesis models, we determined that *Ric-8A* genetic ablation attenuated the abundance and melanoma-driving potential of Gαq-Q209L. A new conditional *Ric-8A*^Flox/Flox^; *Rosa-CreER*^+/^^−^ mouse strain was derived and used as a tissue source to culture an immortalized, tamoxifen-inducible *Ric-8A* knockout melanocyte cell line that required 12-*O*-tetradecanoylphorbol-13-acetate (TPA, phorbol ester) for growth. The cell line failed to grow tumors when grafted into immune-compromised mice regardless of *Ric-8A* expression. Stable expression of human *GNAQ*^Q209L^, but not *GNAQ*^WT^ in the cell line promoted TPA-independent cell proliferation, and upon cell grafting in mice, the initiation and robust growth of darkly-pigmented melanoma tumors. Deletion of *Ric-8A* in *GNAQ*^Q209L^ cells restored TPA-dependent growth, reduced Gαq-Q209L below detectable levels and completely mitigated tumorigenesis from primary or secondary cell line grafts. Interestingly, TPA treatment of cultured *GNAQ*^Q209L^ cells or host animals grafted with *GNAQ*^Q209L^ cells also sharply reduced Gαq-Q209L abundance and tumorigenic capacity. Finally, tumorigenesis initiated from *GNAQ*^Q209L^ cell grafts, followed by host mouse systemic tamoxifen treatment to delete *Ric-8A* in the grafted cells completely abrogated *GNAQ*^Q209L^-driven tumor progression unless a stable human *RIC-8A* transgene was used to rescue the floxed *Ric-8A* alleles. Our work defines two new rational targets that may be developed as potential uveal melanoma therapies through reduction of Gαq/11-Q209L oncoprotein abundance: (1) Ric-8A inhibition and (2) phorbol ester treatment.

## Introduction

Uveal melanomas arise from melanocytes contained in the uveal tract of the ocular cavity. Owing to limited responses to systemic chemotherapies, metastatic forms of the cancer have a poor prognosis with a median survival rate of 12 months, accounting for ~5% of all lethal melanoma cases.^1^ Metastases occur in ~90% of uveal melanoma patients, predominantly to the liver, and to a lesser extent, the lung and bones.^2^ The genetic alterations in uveal melanoma are distinct from cutaneous melanoma, which commonly carry driver mutations in *BRAF* or *NRAS*.^3, 4, 5, 6^ Uveal melanomas are predominantly (⩾85%) driven by mutations in *GNAQ* or *GNA11*, genes that encode the partially redundant heterotrimeric G protein α subunits, Gαq and Gα11.^3, 7, 8, 9, 10^ The point mutations are restricted to residues Q209 and R183, which are critical for intrinsic GTP hydrolysis (GTPase) activity of the G proteins, resulting in persistently active GTP-bound Gα subunits, and therefore constitutively-active signaling.^3, 7, 8, 9^ Q209 mutations are more prevalent than R183 mutations in uveal melanoma because Gαq/11-Q209X proteins are stronger activators of downstream signaling.^8, 11^ Gαq/11-Q209X has poorer GTP hydrolytic activity in comparison with Gαq/11-R183X and is insensitive to regulators of G protein signaling (RGS)-stimulated GTP hydrolysis.^12, 13, 14^

Interestingly, *GNAQ or GNA11* activating mutations induce dermal hyperpigmentation, and are frequently found in cutaneous benign blue nevi and a small subset of melanomas, indicating that overactive Gαq/11 signaling may also be important during priming events of dermal melanocyte neoplasms.^6, 8, 9, 15, 16^ Studies using genetic or xenograft mouse models demonstrated that melanocyte-specific *GNAQ or 11* Q209L expression promoted invasive and metastatic melanoma.^8, 17, 18^ Moreover, massive metastatic cutaneous melanomas were induced when the Gq/11-coupled GPCRs, mGluR1 or mGluR5 were ectopically expressed from mouse melanocyte-specific promoters.^19, 20, 21^ Both mGluRs 1 and 5 have reasonably high basal ability to activate Gq/11 in the absence of agonist.^22, 23^ The emerging evidence is quite convincing that aberrant stimulation of Gαq/11 signaling pathways by hyperactive GPCRs or oncogenic *GNAQ/11* mutations, contributes to the development of various melanocyte neoplasms including cellular transformation and uveal melanoma.^9^

There are no current drugs that directly target oncogenic Gαq/11 proteins, although encouraging developmental efforts are underway.^24, 25^ Existing small-molecule Gαq inhibitors exhibit efficacy to inhibit wild-type Gαq or Gq, and some related G proteins, but fail to attenuate Gαq-Q209L, or provide limited inhibition of Gαq-R183C-dependent signaling in cultured cells.^26, 27, 28^ These inhibitors may ultimately prove useful to block pathogenic, hyperactive GPCR signaling, but the inability to inhibit oncogenic Gαq/11 directly has prompted us to explore an alternative means to block oncogenic Gα subunits by targeting the highly-substrate-specific molecular chaperones, Ric-8A or Ric-8B that act collectively to maintain the proper abundances of all heterotrimeric G protein α subunits.^29, 30, 31^ Studies using cell-free protein translation/folding systems demonstrated that Ric-8A directly participates in the biosynthetic folding of oncogenic Gαq-Q209L and Gαq/i/13.^32^ When these G proteins are produced in the absence Ric-8A, they are mis-folded and rapidly degraded, accounting for massive ~90–95% reductions in membrane-associated G protein levels.^29^

Here, we conducted a proof-of-concept investigation demonstrating that genetic ablation of *Ric-8A* blocked *GNAQ*^Q209L^-driven melanocyte transformation and melanoma pathogenesis using cell graft mouse tumor models. A new C57Bl6J mouse with floxed *Ric-8A* alleles was derived for the work that permitted conditional *Ric-8A* deletion. Primary melanocytes were cultured from this strain and used to create immortalized murine melanocyte cell lines that stably expressed human *GNAQ*^WT^ or oncogenic *GNAQ*^Q209L^. The *GNAQ*^Q209L^ but not the *GNAQ*^WT^ melanocyte cell line exhibited phorbol ester-independent proliferation in culture; a feature associated with melanocyte transformation, and one that was abolished by *Ric-8A* deletion.^33^ When the *GNAQ*^Q209L^ melanocyte cell line was grafted into immune-compromised mice, pigmented melanoma tumors grew robustly. No tumors formed from *GNAQ*^WT^ melanocyte cell line grafts. Deletion of *Ric-8A* in culture before *GNAQ*^Q209L^ cell grafting completely abrogated tumor growth. *Ric-8A*^Flox/Flox^; *GNAQ*^Q209L^ murine melanoma cell lines were cultured *ex vivo* from primary tumor explants and secondary tumor formation from these cells was also blocked by *in vitro Ric-8A* deletion. *GNAQ*^Q209L^ melanocyte cell grafts were then permitted to initiate tumorigenesis, followed by host mouse tamoxifen treatment to delete floxed *Ric-8A* in the grafted melanocytes. Systemic tamoxifen treatment specifically abrogated *GNAQ*^Q209L^-driven tumorigenesis from *Ric-8A*^Flox/Flox^ melanocytes.

We also made an unexpected observation that culture of *GNAQ*^Q209L^ melanocytes in the presence of phorbol ester led to a dramatic decrease in Gαq-Q209L oncoprotein levels. Accordingly, phorbol ester-pre-cultured *GNAQ*^Q209L^ melanocytes completely failed to form melanoma tumors when grafted into mice, and systemic phorbol ester treatment of host mice grafted with *GNAQ*^Q209L^ melanoma cells suppressed tumor initiation and progression. In sum, our study has identified Ric-8A inhibition and phorbol ester (over)stimulation of protein kinase C (PKC) as two new rationale means to attenuate Gαq-Q209L oncoprotein levels. Successful future development of therapeutics against these new targets could provide wanted therapies for *GNAQ/11*-driven uveal melanoma, and perhaps additional oncogenic G protein-influenced cancers.

## Results

### Generation of a conditional *Ric-8A* mouse

Germline deletion of mouse *Ric-8A* causes embryonic lethality due to severe gastrulation defects.^29, 34^ We created a C57Bl/6 J mouse strain with potential for conditional *Ric-8A* knockout using *Ric-8A* gene-targeted embryonic stem cell lines available from the Knockout Mouse Project (KOMP, #CSD70793) (Supplementary Figure S1). *Ric-8A* mice with two copies of floxed exon 5 were viable, reproductive and had no obvious defects; findings consistent to those obtained with a conditional *Ric-8A* mouse produced by a distinct gene targeting strategy.^35^ Mouse embryonic fibroblasts (MEFs) were cultured from our *Ric-8A*^Flox/Flox^ mice and infected with a Cre-NLS-expressing lentivirus. PCR analysis of MEF genomic DNA revealed efficient Cre-mediated deletion of *Ric-8A* exon 5 (Supplementary Figure S2). Immunoblot analyses of MEF lysates demonstrated efficient Cre-mediated reduction of Ric-8A and concomitant reductions in the steady-state levels of heterotrimeric G protein α subunits folded by Ric-8A (Supplementary Figure S2).^32^

### Generation of a mouse melanocyte cell line with potential to conditionally delete *Ric-8A*

**Figure 1:**
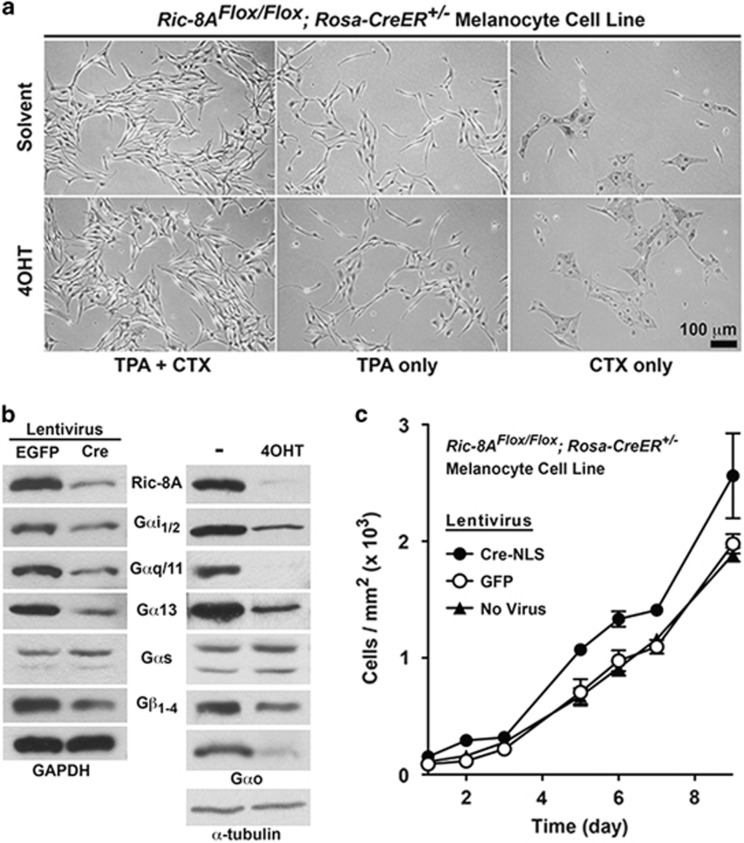
Deletion of *Ric-8A* in murine melanocytes confers a modest cell proliferation advantage, but does not confer TPA- and/or CTX-independent growth. (**a**) Bright-field images of untreated or 4OHT-treated immortalized *Ric-8A*^Flox/Flox^; *Rosa-CreER*^*+/*^^−^ melanocytes grown in the presence of CTX, or TPA, or both for 4 days. (**b**) Quantitative western blot analyses of Ric-8A, Gαi_1/2_, Gαq/11, Gα13, Gαs, Gαo and Gβ_1−4_ levels in Cre or GFP (control) lentivirus, or 4OHT-treated and untreated cultured melanocyte cell lines. Relative GAPDH or α-tubulin levels are shown as normalization controls. (**c**) Cell proliferation analyses of untransduced, and Cre or GFP (control) lentivirus-infected *Ric-8A*^Flox/Flox^; *Rosa-CreER*^*+/−*^ melanocytes. Error bars are the mean±s.e.m. of experiments performed in triplicate.

To investigate the effect of *Ric-8A* deletion on Gα subunit abundances in melanocytes and melanocyte transformation induced by oncogenic Gαq-Q209L, we first created and characterized an immortalized melanocyte cell line with inducible *Ric-8A* knockout potential. Primary melanocytes were isolated from *Ric-8A*^Flox/Flox^; *Rosa-CreER*^+/−^ neonatal mice and immortalized by serial passaging in medium containing 12-*O*-tetradecanoylphorbol-13-acetate (TPA, a phorbol ester) and cholera toxin (CTX).^36, 37^ The *Ric-8A*^Flox/Flox^; *Rosa-CreER*^+/−^ melanocyte cell line exhibited TPA- and CTX-dependent growth, a shared characteristic with the non-tumorigenic mouse melanocyte cell line, Melan-a (Figure 1a).^36^

Ric-8A is produced in melanocytes, as are tested examples from all four heterotrimeric G protein α subunit classes (Gαi/Gαo, Gαq, Gα13 and Gαs). *Ric-8A* deletion in the melanocyte cell line mediated by Cre-NLS lentiviral infection or by 4-hydroxytamoxifen (4OHT) activation of Cre recombinase, decreased Ric-8A abundance and caused concomitant decreases in the levels of G protein subunits folded by Ric-8A (Figure 1b). Gαs levels remained unchanged because this subunit is folded by Ric-8B.^29, 32, 38^

*Ric-8A* deletion had no effect on the requirements of TPA or CTX for melanocyte cell line growth, but did modestly enhance the cell proliferation rate (Figures 1a and c). Enhanced proliferation has been observed for other *Ric-8A* null cell types.^29, 39^ We hypothesize that *Ric-8A* deletion and the consequent decreases in G protein α subunit abundances (Gαq/i/13 classes) release a modest cell proliferation damper conferred normally by homeostatic G protein signaling.

### *Ric-8A* deletion suppresses phorbol ester-independent growth of *GNAQ*^Q209L^-transformed melanocytes

**Figure 2:**
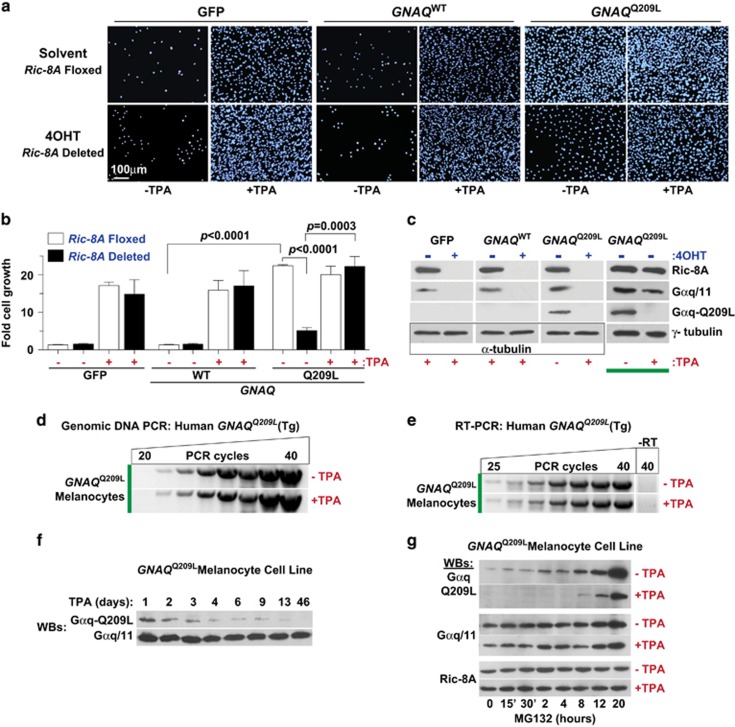
Phorbol ester-independent growth of cultured melanocyte cell lines is conferred by oncogenic *GNAQ*^Q209L^ and reversed by induced *Ric-8A* knockout. (**a**, **b**) Cultured *Ric-8A*^Flox/Flox^; *Rosa-CreER*^*+/*^^−^ melanocyte cell lines stably expressing IRES-GFP, *GNAQ*^WT^-IRES-GFP or *GNAQ*^Q209L^-IRES-GFP were treated with solvent or 4OHT to induce *Ric-8A* knockout, followed by growth in culture medium±TPA for 7 days as indicated. Cells were stained with DAPI and counted using Image J, and the data were reported as fold cell growth. Error bars are the mean±s.e.m. of experiments performed in triplicate. Student's *t*-tests were used to denote significant differences. (**c**) Quantitative immunoblot analyses of relative Ric-8A, Gαq/11 and Gαq-Q209L levels in membrane fractions prepared from melanocyte cell lines treated with solvent or 4OHT, ±TPA as indicated. Relative α- or γ-tubulin levels were measured as loading controls. (**d**) Semi-quantitative genomic DNA PCR analysis of the stably integrated human *GNAQ*^Q209L^ transgene in the *GNAQ*^Q209L^-IRES-GFP mouse melanocyte cell line after culture for 11 days±continuous TPA treatment. (**e**) Semi-quantitative RT–PCR analyses of human *GNAQ*^Q209L^ transcript levels from cDNA prepared from the *GNAQ*^Q209L^-IRES-GFP melanocyte cell line after 11 days culture±continuous TPA treatment. (**f**) Quantitative western blots of relative Gαq-Q209L and Gαq/11 levels in crude membrane fractions prepared from the *GNAQ*^Q209L^-melanocyte cell line cultured in the presence of 200 nm TPA for the indicated time course. (**g**) Time course of MG132 proteasome inhibition-mediated recovery of Gαq-Q209L levels in the *GNAQ*^Q209L^-melanocyte cell line cultured continuously in the presence or absence of TPA. Quantitative western blots show relative Gαq-Q209L, Gαq/11 and Ric-8A levels over the 20-h MG132 time course.

Cultured melanocytes, Melan-a cells and our *Ric-8A*^Flox/Flox^; *Rosa-CreER*^+/−^ melanocyte cell line require continuous signaling stimulus of PKC (TPA, a phorbol ester) and Gαs (CTX) for *in vitro* proliferation (Figure 1a).^36, 37, 40^ The TPA growth requirement is bypassed in melanoma tumor explant cultures or by forced expression of the *HRAS*^*G12R*^ or *BRAF*^V600E^ oncogenes in Melan-a cells.^33, 41, 42^ We transduced the *Ric-8A*^Flox/Flox^; *Rosa-CreER*^+/−^ melanocyte cell line with bicistronic lentiviruses expressing human *GNAQ*^Q209L^-*IRES-GFP*, *GNAQ*^WT^-*IRES-GFP* or *IRES-GFP* and selected for stable transgene expression (Supplementary Figure S3). The *GNAQ*^Q209L^ oncogene conferred sustained cell proliferation in the absence of TPA, whereas the *GNAQ*^WT^ and GFP cell lines failed to grow unless TPA was provided (Figures 2a and b).

We next determined that 4OHT-induced *Ric-8A* deletion had no significant effect on *GNAQ*^Q209L^, *GNAQ*^WT^ or GFP melanocyte cell line proliferation when the cells were grown in the presence of TPA (Figure 2b). The 4OHT treatments substantially reduced Ric-8A, Gαq-Q209L oncoprotein and endogenous Gαq/11 levels (Figure 2c). *Ric-8A* deletion dramatically attenuated TPA-independent growth of the *GNAQ*^Q209L^ melanocyte cell line (Figures 2a and b). Ric-8A is required for Gαq-Q209L biosynthetic protein folding.^32^ *Ric-8A* deletion in the melanocyte cell line sharply reduced Gαq-Q209L levels, thereby reversing the TPA-independent growth conferred by this constitutively-active oncoprotein.

Intriguingly, culture of the *GNAQ*^Q209L^ melanocyte cell line in the presence of TPA also resulted in complete downregulation of the Gαq-Q209L oncoprotein (Figure 2c, compare lanes 7 and 8, highlighted by the thick bar). The human *GNAQ*^Q209L^ transgene remained stably integrated in the mouse melanocyte cell line upon extended culture in TPA-containing medium (Figure 2d), and the *GNAQ*^Q209L^ transcript was produced seamlessly as evidenced by RT–PCR analysis (Figure 2e), and visualized by efficient production of GFP from this bicistronic IRES transcript (Supplementary Figure S3). The combined action of constitutively-active Gαq-Q209L signaling and TPA activation of PKC could overstimulate this common signaling pathway, resulting in a cytotoxicity that is overcome by feedback reduction of Gαq-Q209L abundance. TPA-mediated Gαq-Q209L protein reduction in the parental melanocyte cell line begins rapidly at 24 h of treatment, but persists as a chronic effect that slowly diminishes Gαq-Q209L levels over weeks in culture (Figure 2f). Gαq-Q209L exists predominantly in the GTP-bound conformation, and we suspected that Gαq-Q209L-GTP is more susceptible to the cellular protein degradation machinery, in comparison with wild-type Gαq/11, because it is unlikely to be bound to heterotrimeric G protein βγ subunits. The parental GNAQ^Q209L^ melanocyte cell lines cultured persistently in the presence or absence of TPA were treated with the proteasome inhibitor, MG132 over a 20-h time course. A striking recovery of Gαq-Q209L oncoprotein was observed upon MG132 treatment, whereas wild-type Gαq/11 and Ric-8A levels hardly fluctuated (Figure 2g). Importantly, the degree of MG132-mediated recovery of Gαq-Q209L levels in the chronically-treated TPA cell line was substantially less than that in the TPA-untreated cell line. These results suggest that phorbol esters may present a viable means to reduce Gαq-Q209L oncoprotein levels and attendant cancer-driving signaling. For experimental considerations, TPA exclusion from melanocyte cell culture medium is required to maintain Gαq-Q209L oncoprotein levels. TPA inclusion is required for growth of all other melanocyte cell lines that lacked the *GNAQ*^Q209L^ oncogene.

### Phorbol ester- or *Ric-8A* deletion-induced Gαq-Q209L abundance reduction suppresses *GNAQ*^Q209L^-driven melanoma tumorigenesis

**Figure 3:**
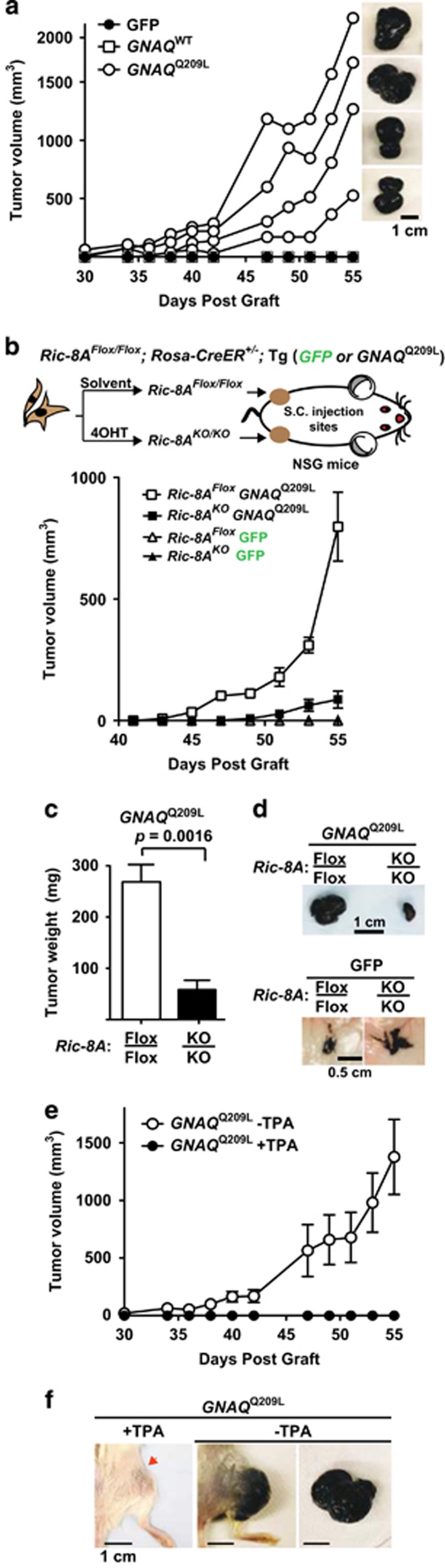
Attenuation of melanocyte Gαq-Q209L levels by *Ric-8A* deletion or phorbol ester treatment blocks *GNAQ*^Q209L^-driven melanoma tumor progression in engrafted mice. (**a**) Tumor growth kinetics of subcutaneously grafted *Ric-8A*^Flox/Flox^; *Rosa-CreER*^*+/−*^ melanocyte cell lines that stably expressed GFP, *GNAQ*^WT^ or *GNAQ*^Q209L^. Images of *GNAQ*^Q209L^ tumors excised at the termination of the experiment are shown alongside each individual growth rate trace. (**b**) Tumor progression kinetics of GFP-expressing or *GNAQ*^Q209L^-transformed *Ric-8*^Flox/Flox^; *Rosa-CreER*^*+/−*^ melanocyte cell lines that were untreated or 4OHT treated to induce *Ric-8A* deletion before subcutaneous engraftment. (**c**) Average weights of *Ric-8A*^Flox/Flox^ or *Ric-8A*^*KO/KO*^*GNAQ*^Q209L^-driven melanoma tumors at the experimental end point of 59 days. (**d**) Representative images of isolated *GNAQ*^Q209L^-driven tumors and the mouse injection sites of the GFP melanocyte cell line that showed no signs of tumor growth at day 59 post injection. Error bars are the mean±s.e.m. of 3–4 independent experiments. (**e**) Tumor growth kinetics of subcutaneously grafted *Ric-8A*^*Flox/Flox*^*; Rosa-CreER*^*+/−*^; Tg (*GNAQ*^Q209L^-IRES-GFP) melanocyte cell line pre-cultured in the presence or absence of TPA. (**f**) Representative images of excised tumors obtained from grafted *GNAQ*^Q209L^ cells that had been pre-cultured without TPA and a representative subcutaneous injection site of grafted *GNAQ*^Q209L^ cells that had been pre-cultured in the presence of TPA at the termination of the experiment (day 55). Data are the mean±s.e.m. (*n*=4).

The *GNAQ*^Q209L^, *GNAQ*^WT^ and the GFP (*Ric-8A*^Flox/Flox^; *Rosa-CreER*^+/−^) melanocyte cell lines (5 × 10^6^ cells each) were grafted subcutaneously into the rear flanks of immune-compromised NSG mice. *GNAQ*^Q209L^ cells pre-cultured in the absence of TPA formed tumors that could first be measured beneath the skin after an ~28-day latency. The tumors continued to grow for an additional ~30 days until the mice were killed and the heavily melanin-pigmented tumors were excised and weighed. The *GNAQ*^Q209L^ tumors were roughly 0.6–2.0 cm in diameter and often oblong and/or multi-lobed. No tumors were formed from *GNAQ*^WT^ or GFP cell grafts (Figure 3a).

The *GNAQ*^Q209L^ and control GFP melanocyte cell lines were then treated with or without 4OHT in culture to delete *Ric-8A* before grafting into NSG mice (left flanks, solvent-treated cells and right flanks, 4OHT-treated cells). The GFP-only melanocyte cell line had no tumorigenic capacity regardless of *Ric-8A* expression or deletion (Figures 3a and d, bottom panel). *Ric-8A* deletion substantially attenuated *GNAQ*^Q209L^-driven tumor progression (Figure 3b) and measured tumor weights at the completion of the experiments (Figures 3c and d). This indicates that the modest *in vitro* melanocyte proliferation advantage conferred by *Ric-8A* knockout (Figure 1c) was negated by loss of Gαq-Q209L oncoprotein folding capacity during *in vivo* tumor growth.

*GNAQ*^Q209L^ cells pre-cultured in the presence of TPA also had dramatically reduced Gαq-Q209L levels (Figure 2c) and accordingly, failed to grow melanoma tumors when grafted into mice (Figures 3e and f). PKC overstimulus by the combined action of Gαq-Q209L-stimulated diacylglycerol (DAG) production and the exogenous DAG mimetic, TPA may induce a melanocyte cytotoxicity that is overcome by Gαq-Q209L protein downregulation. This raises the enticing possibility that phorbol esters may be used therapeutically to induce cancer cell toxicity in *GNAQ/11*-driven uveal melanomas.

### *Ric-8A* deletion suppresses secondary tumor progression of grafted, *ex vivo* cultured *GNAQ*^Q209L^ melanoma cell lines

**Figure 4:**
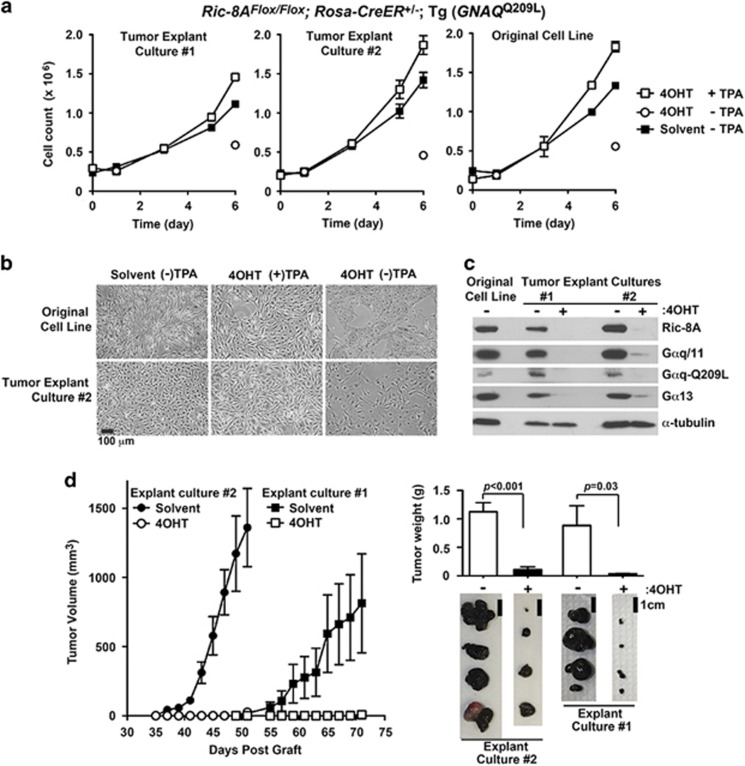
*GNAQ*^Q209L^-driven secondary melanoma tumor progression is attenuated by induced *Ric-8A* deletion. (**a**) Two explanted *GNAQ*^Q209L^ tumors from Figure 3a were cultured *ex vivo* in standard melanocyte culture medium lacking TPA to establish *Ric-8A*^Flox/Flox^; *Rosa-CreER*^+/^^−^; (Tg) *GNAQ*^Q209L^ melanoma cell lines. The growth kinetics of the melanoma cell lines±TPA supplementation and ±4OHT to induce *ex vivo Ric-8A* deletion as indicated were compared with the growth progression of the original *Ric-8A*^Flox/Flox^; *Rosa-CreER*^+/^^−^; (Tg) *GNAQ*^Q209L^ melanocyte cell line. (**b**) Bright-field images of the parental *Ric-8A*^Flox/Flox^; *Rosa-CreER*^+/^^−^; (Tg) *GNAQ*^Q209L^ melanocyte cell line and melanoma explant culture #2 at the conclusion of the 6-day growth study. Error bars are the mean±s.e.m. of experiments performed in triplicate. (**c**) Quantitative western blot analyses of Ric-8A, Gαq/11, Gαq-Q209L and Gα13 levels in lysates prepared from the parental *Ric-8A*^Flox/Flox^; *Rosa-CreER*^*+/−*^; (Tg) *GNAQ*^Q209L^ melanocyte cell line and melanoma cell cultures derived from the two independent explanted *GNAQ*^Q209L^-melanoma tumors. Tumor cell cultures were treated with 4OHT to induce *Ric-8A* knockout *ex vivo*. α-Tubulin levels were measured as a normalization control. (**d**) Secondary tumor progression from grafted *Ric-8A*^Flox/Flox^; *Rosa-CreER*^+/^^−^; (Tg) *GNAQ*^Q209L^ explant cultures #1 and #2 after *in vitro* treatment with solvent or 4OHT to induce *Ric-8A* deletion. Shown alongside are images and average weights of harvested secondary tumors at day 56 (culture #2) and day 71 (culture #1). Error bars are the mean±s.e.m. (*n*=4 tumors).

Excised *Ric-8A*^Flox/Flox^; *Rosa-CreER*^+/−^; Tg (*GNAQ*^Q209L^) tumors from Figure 3a were cultured *ex vivo* in standard melanocyte culture medium lacking TPA to derive two independent *GNAQ*^Q209L^ melanoma cell lines, with which to determine the effect of *Ric-8A* deletion on secondary tumor progression. The melanoma cell lines exhibited TPA-independent growth and acquired TPA dependence after *Ric-8A* deletion, both characteristics of the parental *GNAQ*^Q209L^ melanocyte cell line used to generate the primary tumors (Figures 4a and b). The *GNAQ*^Q209L^ melanoma cell lines retained melanin pigmentation but had adopted a morphology that was more spindle-shaped or epithelial-like in comparison with the parental melanocyte cell line (Figure 4b). Both tumor cell lines retained the capacity to induce *Ric-8A* deletion and deplete endogenous G proteins and Gαq-Q209L (Figure 4c). The melanoma cell lines were pre-treated with 4OHT *ex vivo* to induce *Ric-8A* knockout before secondary graft experiments (Figure 4d). *Ric-8A* knockout dramatically blunted Gαq-Q209L-driven secondary melanoma tumor progression. One tumor cell line exhibited accelerated tumorigenic onset and progression whereas the second cell line exhibited kinetics similar to, or slightly delayed in comparison with the parental *GNAQ*^Q209L^ melanocyte cell line.

### Host animal tamoxifen treatment induces *Ric-8A* deletion in grafted tumor cells and blunts *GNAQ*^Q209L^ tumorigenesis

**Figure 5:**
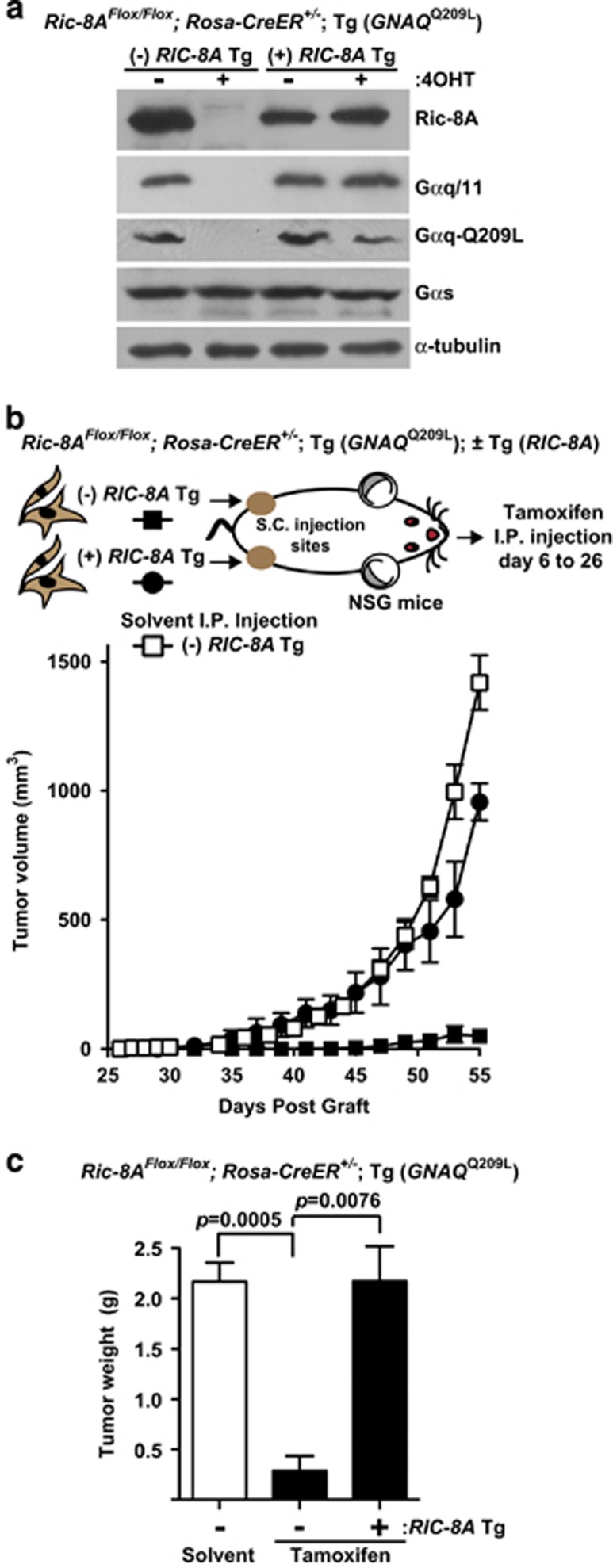
Whole animal tamoxifen treatment deletes *Ric-8A* in grafted cells to block *GNAQ*^Q209L^ melanoma tumor growth. (**a**) Quantitative western blot analyses of Gαq/11, Gαq-Q209L, Gαs, Ric-8A and α-tubulin levels in *GNAQ*^Q209L^-transformed *Ric-8A*^Flox/Flox^; *Rosa-CreER*^+/^^−^ melanocyte cell lines with or without stable expression of a human *RIC-8A* cDNA transgene (Tg). Floxed mouse *Ric-8A* deletion was induced by 4OHT treatment in culture as indicated. (**b**) Tumor growth kinetics in mice grafted with Gαq-Q209L-transformed *Ric-8A*^Flox/Flox^; *Rosa-CreER*^*+/−*^ melanocyte cell lines with or without stable *RIC-8A* Tg expression, followed by intraperitoneal (i.p.) tamoxifen or solvent mouse treatments post grafting to induce *Ric-8A* Flox allele deletion in the grafted cells. (**c**) Average weights of tumors grown from *GNAQ*^Q209L^ melanocyte cell line grafts with or without *RIC-8A* Tg at the termination of the experiment (day 58). Error bars are the mean±s.e.m. (*n*=3–5).

The *Ric-8A*^Flox/Flox^; *Rosa-CreER*^+/^^−^; Tg (*GNAQ*^Q209L^) melanocyte cell line was transduced with a human *RIC-8A* cDNA or control lentivirus and selected for stable expression of the transgene. 4OHT treatment of both melanocyte cell lines in culture induced deletion of the floxed, mouse *Ric-8A* alleles, which resulted in greatly reduced Gαq-Q209L and Gαq/11 levels in the cell line lacking the *RIC-8A* transgene. Gαq-Q209L remained at normal levels in the counterpart *RIC-8A* transgene expressing cell line, despite 4OHT treatment (Figure 5a).

Control and *RIC-8A* transgene cell lines were grafted into the left and right flanks of NSG mice, respectively. At days 6 through 26 post-subcutaneous grafts, the mice were intraperitoneally (i.p.) injected with tamoxifen *q.a.d.* to induce floxed *Ric-8A* deletion in the grafted cells. Solvent injections were performed similarly for control, *Ric-8A*^Flox/Flox^-grafted mice (that is, no *RIC-8A* transgene). *In vivo* tamoxifen treatment effectively ablated tumorigenesis driven by *GNAQ*^Q209L^ in the *Ric-8A*^Flox/Flox^ background, but not in the background in which the floxed *Ric-8A* alleles were rescued by expression of the *RIC-8A* cDNA transgene (Figures 5b and c). These results clearly show that loss of tumor cell *Ric-8A* expression and not whole animal tamoxifen treatment *per se* accounts for the block of *GNAQ*^Q209L^-driven melanoma tumorigenesis.

### Host animal phorbol ester treatment suppresses melanoma tumorigenesis of grafted *GNAQ*^Q209L^ melanocytes

**Figure 6:**
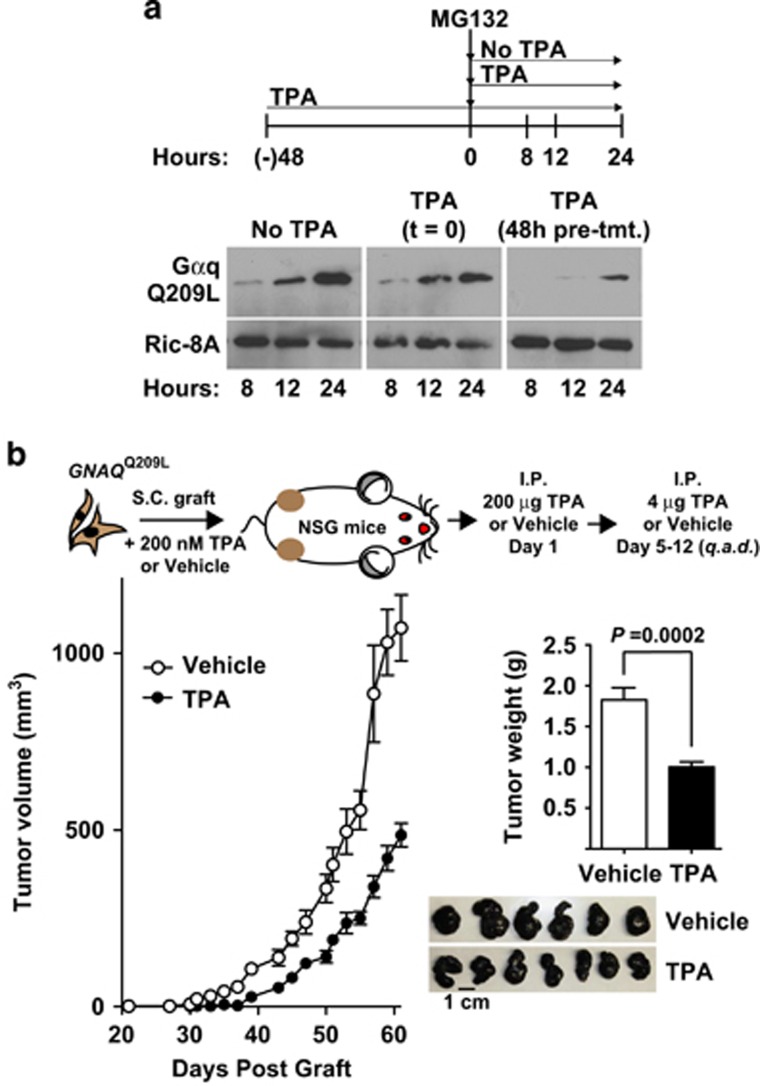
Phorbol ester attenuates cultured cell *GNAQ*^Q209L^ levels and *in vivo* melanoma tumorigenesis. (**a**) Quantitative western blot analyses of Gαq-Q209L and Ric-8A (control) levels in *GNAQ*^Q209L^ melanoma cell line lysates prepared at the indicated times of MG132 treatment. Three TPA treatment schedules were followed: no treatment, co-treatment with MG132 and 48 h pre-treatment and co-treatment with MG132. (**b**) Tumor growth kinetics of NSG mice grafted with the Gαq-Q209L melanocyte cell line, followed by systemic animal treatment with the indicated schedule of TPA or vehicle. The average weights and images of tumors isolated from TPA- and vehicle-treated mice at day 61 post cell grafting. Error bars are the mean±s.e.m. (*n*=6 or 7).

*A Ric-8A*^Flox/Flox^; *Rosa-CreER*^+/^^−^; Tg (*GNAQ*^Q209L^) melanoma cell line was generated from an excised primary tumor in Figure 4 and continuously cultured in the absence of TPA. This cell line was treated acutely with or without TPA for 48 h and then for an additional 24 h in the presence of MG132 ±TPA (Figure 6a). MG132 treatment provided a substantial boost in Gαq-209L abundance, as shown for the parental *GNAQ*^Q209L^ melanocyte cell line in Figure 2g. The acute TPA treatment markedly reduced Gαq-Q209L levels, as well as the ability of MG132 to provide recovery, demonstrating that the established melanoma cell line exhibits *in vitro* responsiveness to phorbol ester-mediated Gαq-Q209L oncoprotein level reduction (Figure 6a). Finally, the prospective ability of phorbol ester to suppress Gαq-Q209L-driven tumorigenesis *in vivo* was measured following subcutaneous grafting of *GNAQ*^Q209L^ cells and systemic treatment of the host animal with TPA as outlined in the schedule of Figure 6b. TPA-treated host animals had markedly delayed tumor onset and a modestly reduced progression rate in comparison with vehicle-treated animals. Overall, these results collectively demonstrate two future possibilities to amerliorate *GNAQ/11*-induced uveal melanoma; reduction of Gαq/11-Q209L driver oncoprotein levels through Ric-8A inhibition or phorbol ester treatment.

## Discussion

The present study provides a genetic demonstration that melanocyte deletion of the molecular chaperone Ric-8A suppressed tumorigenesis mediated by the uveal melanoma oncogenic driver G protein, Gαq-Q209L. The means of inhibition was to deplete cellular oncoprotein levels below a threshold required to manifest hyperactive, cancer-driving signaling. Our intent with this work is to provide proof-of-concept that a properly developed Ric-8A inhibition strategy may ultimately be used to block onco-G protein-driven cancers. Therapeutic Ric-8A inhibition could be extended to GPCR-driven diseases where depletion of endogenous G proteins might be more efficacious in comparison with antagonism of select GPCR(s). Blunting the abundance of client oncoproteins through inhibition of the chaperones that fold them is an active strategy in cancer therapeutic development, with Hsp90 inhibitors providing a prominent precedent.^43, 44^ Blocking the highly specialized chaperones Ric-8A or B, that to date are known to only fold subsets of G protein α subunits, represents an untested target against onco-G-protein-driven cancers. Uveal melanoma is perhaps the clearest example in which the oncoprotein driver is a G protein, Gαq/11-Q209L, which is folded by Ric-8A.

For this work, a new C57Bl/6 J transgenic mouse strain with inducible *Ric-8A* knockout potential was derived and used as a source to create immortalized melanocyte cell lines from neonatal dermal explants. The parental melanocyte cell line was made to stably express human *GNAQ*^WT^ or *GNAQ*^Q209L^. Induced *Ric-8A* deletion in the cultured melanocyte cell lines effectively reduced Gαq-Q209L protein levels and had no obvious cytotoxic effects. In fact, *Ric-8A* deletion imparted a modest *in vitro* growth advantage to cultured melanocytes and melanoma cell lines. This effect has been observed for other *Ric-8A-*null cell types and suggests that the net effect of endogenous G protein signaling is to provide a cell proliferation brake.^29, 39^ However, this *in vitro* growth enhancement provided no tumorigenic propensity when *Ric-8A*-null cells lacking a driver oncogene were grafted into immune-compromised mice. *Ric-8A* deletion completely blocked melanoma tumorigenesis of the grafted *GNAQ*^Q209L^ melanocyte cell line and secondarily-derived *GNAQ*^Q209L^ melanoma cell lines.

A potential complication of any approach to genetically disrupt or inhibit Ric-8A is the reduction in endogenous G protein levels that will occur. On the other hand, if Ric-8A inhibition could be directed to tumor cells, the effect on endogenous G proteins may provide added therapeutic efficacy of tumor inhibition. G protein signaling imparts important advantages within the tumor microenvironment including pro-migratory properties of cancer cells, secretion of angiogenic factors that promote tumor vascularization, responsiveness to host growth factors and usurpation of the immune system. *Ric-8A* deletion/inhibition-mediated reduction of endogenous G proteins would potentially mitigate all of these processes. An additional observation made with *Ric-8A* null cells may be specifically relevant to a potential uveal melanoma therapy; Gαq/11-Q209L-mediated uveal melanoma progression involves constitutive stimulation of Rho/Rac guanine nucleotide exchange factors (GEFs) that regulate actin polymerization, which is thought to contribute the signal that activates YAP as the main signaling driver of the cancer.^17, 45^ *Ric-8A*-null mouse embryonic stem cells and *Ric-8A* shRNAi-treated MEFs have substantial deficiencies in polymerized actin levels (F-actin) that may be attributable to reductions in the G proteins (Gα12/13/q/11) primarily responsible for stimulating Rho-signaling pathways.^29, 39^ So, in addition to inhibited Ric-8A resulting in reduced Gαq/11-Q209L oncoprotein levels, there could be additional attenuation of the YAP pathway at the level of reduced actin polymerization.

Our findings help clarify a current controversy regarding Ric-8 cellular activity. Ric-8A was initially characterized as a GEF that stimulates Gα subunit guanine nucleotide exchange *in vitro*.^46^ Evidence from many organismal systems showed that Ric-8 orthologs are required to maintain proper G protein abundances.^29, 30, 31^ Ric-8 acts as a chaperone during biosynthesis to facilitate Gα subunit protein folding. G proteins produced in the absence of Ric-8 are mis-folded and rapidly degraded.^29, 32^ It is not clear whether GEF and chaperoning activities are one and the same, or whether Ric-8 is a multi-functional protein that acts during biosynthetic G protein folding and later facilitates Gα GTP binding to evoke signaling outputs. The fact that *Ric-8A* deletion suppresses the oncogenic action of Gαq-Q209L indicates that the chaperoning activity is either the authentic cellular activity or is more penetrant than GEF activity. The Gαq/11-Q209L oncoprotein has greatly impaired GTP hydrolysis activity that renders it constitutively active, therefore bypassing the need of a GEF for sustained signaling output.

A key remaining question is to understand why *GNAQ/11*^Q209L^ drives the majority of uveal melanomas, yet the oncogene is far less prevalent in cutaneous melanomas.^6, 8, 9^ Deciphering this difference will provide useful insight toward the development of melanoma therapies. We hypothesize that uveal melanocytes possess a survival privilege stemming from an innate ability to tolerate a higher ‘dose' of Gαq/11 signaling, or as others have suggested, ocular melanocytes are compartmentally shielded from attack by the host immune system.^47^ Cutaneous melanocytes may be more sensitive to Gαq/11 signaling and have a capacity to downregulate *GNAQ/11*^Q209L^ transcript levels or Gαq/11-Q209L protein levels, or otherwise enter into an apoptotic cell death program.^18^ Constitutively-active *GNAQ/11* mutations are in fact highly prevalent in benign melanocytic neoplasms and can cause skin hyperpigmentation.^7, 8, 9, 18^ These pre-cancerous dermal lesions could require a lower threshold of Gαq/11 signaling to develop, yet the higher level necessary to drive cellular transformation may not be well tolerated by dermal melanocytes. There are many clear examples in which modestly elevated Gq/11 signaling induces proliferation in specific tissues or cultured cell lines, but chronic Gq/11-coupled GPCR agonist treatment(s) or constitutively-active mutant overexpression induces apoptosis.^48, 49, 50, 51, 52^

In accordance with this hypothesis, when our *GNAQ*^Q209L^ cutaneous murine melanocyte or melanoma cell lines were treated in culture with a phorbol ester, Gαq-Q209L protein abundance, but not wild-type Gαq/11 abundance was reduced dramatically, an effect on the order of that which occurred when *Ric-8A* is deleted. The TPA-treated *GNAQ*^Q209L^ cell line did not form tumors when grafted, despite efficient production of the *GNAQ*^Q209L^-IRES-GFP transcript as visualized via the efficient GFP fluorescent signal (Supplementary Figure S3) and by RT–PCR analyses (Figure 2e). We suspect that overstimulation of the phospholipase Cβ DAG/ PKC branch of the Gq/11 signaling axis is responsible for a feedback pathway that can reduce Gαq/11 oncoprotein levels in dermal melanocytes. TPA is a DAG mimetic that activates PKC. Gαq/11 oncoprotein cancer signaling is thought to be primarily driven by the distinct Rho/YAP branch of the Gq/11 signaling axis.^17, 45^ When oncoprotein levels are reduced by feedback inhibition through PKC, cancer signaling through Rho/YAP would also be reduced, potentially explaining the great difference in frequency of cutaneous versus uveal melanomas driven by oncogenic *GNAQ/11*. We are actively investigating the potentially distinct signaling properties of ocular and cutaneous melanocytes to cipher out an explanation of why *GNAQ/11*^Q209L^-induced oncogenesis is highly biased toward uveal melanocytes.

Our results raise the intriguing prospect that phorbol esters may be an effective way to mitigate uveal melanoma oncogenesis. Phorbol esters directly activate PKC and are commonly thought of as tumor promoters. Yet for *GNAQ/11*^Q209L^-driven uveal melanoma, super-activation of the Gαq/11-Q209L-stimulated PKC pathway could provide two distinct mechanisms of therapy: (1) PKC-activated feedback reduction of Gαq/11 oncoprotein abundance or (2) inducement of cancer cell apoptosis. PKC activators, including phorbol esters, inhibited growth of various non-uveal melanoma cell lines and tumors through inducement of cell-cycle arrest or apoptosis.^53, 54, 55, 56^ Wild-type *PKC* rescue of loss-of-function *PKC* alleles in human tumor cells inhibited tumorigenesis, showing that PKC is a tumor suppressor.^57^ Our demonstration in Figure 6b that host animal systemic TPA treatment delayed melanoma tumor onset and progression from a grafted *GNAQ*^Q209L^ cell line is highly encouraging. Our ongoing and future work involves active trials to reduce Gαq/11-Q209L oncoproteins through Ric-8A inhibition or phorbol ester inhibitory feedback as two new potential strategies to treat *GNAQ/11*^Q209L^ melanoma.

## Materials and methods

### Creation of a conditional *Ric-8A* knockout mouse

The *Ric-8A* knockout-first allele targeted mouse embryonic stem cell (ES) lines were purchased from Knockout Mouse Project (KOMP) Repository (Project ID CSD70793) at UC Davis. Microinjection and implantation of *Ric-8A-Neo* targeted ES cells was carried out by the Gene Targeting and Transgenic Mouse core facility at the University of Rochester Medical Center. Chimeras were mated with C57Bl/6 J mice (Jackson Lab, Bar Harbor, ME, USA, stock # 000664). One out of four chimeras carried a germline copy of the *Ric-8A*-*Neo* allele. Mice harboring the *Ric-8A Neo* allele were crossed with FLPer mice (Jax Lab stock # 009086) to generate *Ric-8A floxed* allele progeny. *Ric-8A* homofloxed mice that express tamoxifen-inducible-Cre (CreER) recombinase driven by the ubiquitous Rosa promoter were generated upon breeding to R26-Cre-ER^T2^ mice (Jax Lab stock # 008463). Mice were handled and maintained in accordance with University of Rochester Institutional Animal Care and Use committee.

### Cell culture

MEFs were isolated from day E13.5 *Ric-8A*^Flox/Flox^ embryos and cultured in DMEM containing 10% FBS.^58^ Spontaneously immortalized melanocyte cell lines were generated from epidermal cell explants isolated from 3-day old neonates as described.^36^ In detail, cell explants were co-cultured in melanocyte medium (RPMI-1640, 10% FBS, 2 mm l-Glutamine, 100 U/ml penicillin, 100 μg/ml streptomycin, 200 nm TPA, 200 pm CTX and 200 μm phenylthiourea) with XB2 keratinocyte feeder cells (ATCCCL-177, ATCC, Manassas, VA, USA) that had been mitotically-inactivated with mitomycin C. Growth of melanocytes was selectively promoted by TPA and CTX supplementation.^36, 37^ After passage 4, XB2 feeder cells were eliminated and melanocytes were passaged past the senescence phase until colonies began to grow. Spontaneously immortalized, feeder cell-free, melanocyte cell line growth was confirmed by examination of cells by microscopy for the presence of melanin pigment granules.

MG132 (in DMSO) at 10 μm final concentration in melanocyte medium was used to inhibit proteasome-mediated degradation in *GNAQ*^Q209L^-melanocyte or -melanoma cell line culture. The MG132-treated cells were washed with phosphate-buffered saline with protease inhibitor mixture (23 μg/ml phenylmethylsulfonyl fluoride, 21 μg/ml Nα-p-tosyl-l-lysine-chloromethyl ketone, 21 μg/ml l-1-p-tosylamino-2-phenylethyl-chloromethyl ketone, 3.3 μg/ml leupeptin and 3.3 μg/ml lima bean trypsin inhibitor) and scraped from the culture dish. Cells were pelleted at 1000 *g* for 5 min. Pelleted cells were lysed in the sample buffer containing protease inhibitors mixture and 10 μm MG132, boiled at 95 °C for 5 min and centrifuged at 1 50 000 *g* for 5 min. The resulting supernatants were used for western blot analyses.

To prepare crude membranes, cultured cells were washed and scraped in phosphate-buffered saline with protease inhibitor mixture. The cells were lysed by nitrogen cavitation using a Parr bomb (Parr Instrument Co., Moline, IL, USA). The lysate was clarified by centrifugation at 500 *g* for 5 min. The 500-g supernatant was centrifuged at 1 50 000 *g* for 45 min. The resulting crude membrane pellet was solubilized in sample buffer with protease inhibitor mixture and processed for western blot analyses.

### Molecular cloning

The coding sequence of Cre recombinase was amplified from pENTR4-CreERT2^59^ using the forward primer: 5′-CGCGCGCCATGGATGCCAAAAAAAAAGAGGAAGGTGTCCAATTTACTGACCGTACACC-3′, encoding the N-terminal nuclear localization signal, MPKKKRK, and the reverse primer: 5′-CGCGCGGTCGACCTAATCGCCATCTTCCAGCAGGCG-3′ and inserted into the Gateway Entry Vector, pENTR4.^60^ A bicistronic pENTR4-IRES-GFP vector was created by excision of the IRES-GFP cassette from pIRES2-GFP (gift from Dr David I Yule) with *Sal*I-*Xba*I and ligation into these sites of pENTR4. The coding sequences of human *GNAQ*^WT^ or *GNAQ*^Q209L^ were PCR amplified from the corresponding pcDNA3.1 constructs (cDNA Resource Center) using linker-based oligonucleotides and inserted 5′ of the IRES-GFP cassette in pENTR4-IRES-GFP. Lentiviral donor expression vectors were created using the Gateway LR Clonase Enzyme kit (Life Technologies, Carlsbad, CA, USA) to recombine the Cre-NLS-IRES-GFP, and *GNAQ*^WT^- or *GNAQ*^Q209L^-IRES-GFP modules from the respective pENTR4 entry vectors into the CMV promoter Gateway destination vectors, pLenti-Hygro or Puro.^61^

### Genomic DNA and reverse transcription PCR

PCR was performed using 100 ng of genomic DNA extracted from *GNAQ*^Q209L^ and control melanocyte cell lines using a Qiagen DNAeasy Blood and Tissue kit (Qiagen, Valencia, PA, USA). The following human *GNAQ*-specific primer pair was used, forward primer: 5′-GCGCGCGCGTACGCGCATGACTCTGGAGTCCATCATG-3′ and reverse primer: 5′-CGCGCGCAATTGTTAGACCAGATTGTACTCCTTCAG-3′ with the program: 40 cycles of 94 °C for 1 min, 56 °C for 45 s, 72 °C for 1 min 30 s, followed by a 5-min extension phase at 72 °C. Total RNA was isolated from *GNAQ*^Q209L^ and control melanocyte cell lines that had been treated with or without TPA for 11 days. Total RNA (400 ng) was reverse transcribed using the *GNAQ*^Q209L^-specific antisense primer, 5′-CCATTTTCTTCTCTCTGACCTTAGGCCCCCTACATCGACC-3′ with a Superscript III Reverse transcriptase kit (Thermo Fisher Scientific, Waltham, MA, USA). cDNA was subjected to nested PCR using the human *GNAQ*^Q209L^ transcript-specific primer pair: forward primer 5′-GCGCGCGCGTACGCGCATGACTCTGGAGTCCATCATG-3′ and reverse primer 5′- GGTAGGCAGGGTCAGCTACGC-3′ with the program: 40 cycles of 94 °C for 1 min, 53 °C for 45 s, 72 °C for 1 min 30 s, followed by a 5-min extension phase at 72 °C. PCR products were obtained after the indicated number of amplification cycles and resolved on ethidium bromide-stained 1% Agarose gel and visualized by UV transillumination.

### Lentivirus production and infection

Lentiviruses were prepared from the pLenti-Cre-NLS, pLenti-IRES-GFP and pLenti-*GNAQ*^WT^ or -*GNAQ*^Q209L^-IRES-GFP donor vectors as follows: 4 × 10^6^ HEK293T cells were seeded in 10-cm diameter culture dishes in DMEM containing 10% FBS. After 24 h, cells were transfected with 4 μg of pLenti construct, 2.5 μg of pMDLg/pRRE, 2.5 μg of pRSV-Rev and 2 μg of pSVS/pMD2.g using Lipofectamine 2000 (Thermo Fisher).^62^ Lentiviral-containing media overlaying the transfected cells was harvested at 48 h, filtered through a 0.45-μm syringe filter and supplemented with 8 μg/ml of polybrene (Sigma-Aldrich, St Louis, MO, USA). Immortalized mouse melanocytes were infected with 1:1 ratio of viral medium: fresh melanocyte medium for 48 h. Transduced melanocytes were selected with 200 μg/ml and then 100 μg/ml hygromycin B over 72 h and maintained in melanocyte medium containing 50 μg/ml hygromycin B (Thermo Fisher). The *GNAQ*^WT^ and GFP stable melanocyte cell lines were continuously cultured in the presence of TPA. The *GNAQ*^Q209L^ melanocyte cell line was cultured in the absence of TPA in order to maintain Gαq-Q209L abundance.

### Genetic ablation of *Ric-8A*

*Ric-8A*^Flox/Flox^ MEFs were infected with Cre-NLS-lentivirus for 48 h, selected with 100 μg/ml hygromycin B for an additional 48 h to achieve *Ric-8A* knockout. Cell lysates were harvested for quantitative western blot analyses and mRNA isolation. Deletion of *Ric-8A* in cultured *Ric-8A*^Flox/Flox^; *Rosa-CreER*^*+/-*^ melanocyte cell lines was achieved by treating the cells in culture with 500 nm 4OHT (Sigma-Aldrich) for 5 days or by infection with Cre-NLS-lentivirus for 48 h followed by selection with 2 μg/ml puromycin (Sigma-Aldrich).

### Cell graft melanoma tumorigenesis models

Eight-week-old female NSG (NOD.Cg-*Prkdcscid Il2rgtm1Wjl*/SzJ) mice were from the Jackson laboratory (Stock #005557). For the subcutaneous cell graft tumor model, live melanocytes were suspended by brief trypsinization, washed extensively in melanocyte medium and counted using a hemacytometer after Trypan blue dye exclusion. NSG host mice were injected subcutaneously in each hind flank with 5 × 10^6^ cells of the GFP-, *GNAQ*^WT^- or *GNAQ*^Q209L^-melanocyte cell lines in RPMI base medium, or 1 × 10^6^ *GNAQ*^Q209L^ tumor-derived melanoma cell lines for the secondary tumor growth progression studies. *In vivo* deletion of *Ric-8A* in melanocyte cell lines grafted into NSG mice was achieved by treating host animals with 10–15 i.p. injections of 1 mg tamoxifen or vehicle over a course of 20 days. Tamoxifen was dissolved in ethanol at 100 mg/ml followed by 1:10 dilution in sterile corn oil. The TPA treatment of host mice was performed by mixing melanocytes in a suspension of RPMI base medium and 200 nm TPA or vehicle before cell grafting. At day 1 post graft, one i.p. injection of 200 μg TPA (in 10% ethanol, 90% corn oil) or vehicle was performed, followed by i.p. injection of 4 μg TPA or vehicle, q.a.d, over days 5–12 post cell grafting. Injection sites were monitored thrice weekly for tumor growth. Tumor size was measured using calipers and volume was calculated using the formula: length × width^2^/2.^63, 64^ Mice were killed when measured tumors reached ~1000 mm^3^ and the tumors were isolated from the adhered tissue.

### Tumor explant cultures

Subcutaneous *GNAQ*^Q209L^ primary melanoma tumors were excised from host animals, chopped into small pieces and treated with 0.1% w/v Collagenase A (Roche, Indianapolis, IN, USA) for 5–10 min at 37 °C. The resulting cell suspension was washed twice and cultured in fresh melanocyte medium.

### Cell imaging

Melanocyte cell lines were fixed with 4% paraformaldehyde and stained with DAPI nuclear stain as described.^29^ DAPI-stained images of fixed cells were captured using Metamorph software (Molecular Devices, Sunnyvale, CA, USA) with UPLSAPO × 10 (n.a. 0.35) objective on an Olympus IX70 microscope and an ORCA-ER digital camera (Hamamatsu, Japan). Phase-contrast images were captured with a Nikon Eclipse TS100 inverted microscope (Nikon, Tokyo, Japan), × 10 objective (n.a. 0.25) using an attached Canon EOS Rebel XSI camera (Canon, Tokyo, Japan).

### Immunoblotting

Rabbit polyclonal Ric-8A [1184],^29^ Gαo [S214]^65^ and Gαi_1/2_ [B084]^66^ antisera were described previously. Rabbit polyclonal Gαq/11 [C-19] and Gα13 [H-300] antisera were purchased from Santa Cruz (Dallas, TX, USA). Monocolonal α-tubulin and γ-tubulin were from Sigma-Aldrich. The antibody against the Gαq-Q209L oncoprotein was purchased from NewEast Biosciences, King of Prussia, PA, USA [26328].

### Statistical analysis

Error bars throughout are the mean±s.e.m. Two-tailed Student's *t*-tests were performed as indicated using GraphPad Prism (La Jolla, CA, USA). *P*-values <0.05 were regarded as significant.
