# Supplementary material for: Ric-8A gene deletion or phorbol ester suppresses tumorigenesis in a mouse model of GNAQQ209L-driven melanoma
Source: Oncogenesis. 2016 Jun 27;5(6):e236–. doi: 10.1038/oncsis.2016.45 (PMC4945744; doi:10.1038/oncsis.2016.45)
Supplement: Supplementary Information [file oncsis201645x1.docx]

**Supplementary Figure Legends**

**Supplementary Fig. 1.**

Creation of the *Ric-8A*^Flox/Flox^ mouse strain. **(A)** Schematic of the *Ric-8A* gene targeting, PCR genotyping, and conditional and knockout allele generation strategies. *Ric-8A* gene-targeted embryonic stem cells were purchased from the Knockout Mouse Project Consortium (KOMP Project # CSD70793 ). Intron 4 of the *Ric-8A* knockout first, *Lac*Z-*Neo* allele is flanked by Flp-recombinase targetable sites (Frt). Exon 5 is flanked by *loxP* sites. Microinjection and implantation of *Ric-8A-LacZ-Neo*-targeted ES cells into host albino blastocysts produced four chimeras, one of which conferred germ-line transmission of the *Ric-8A* *Lac*Z-*Neo* allele. The presence of the transcription inhibitory *Lac*Z-*Neo* cassette would only allow production of a truncated *Ric-8A* mRNA. Therefore, the cassette was removed by Flp recombinase action to enable expression from the full length, floxed conditional *Ric-8A* allele (1). The *Ric-8A Lac*Z-*Neo* germ line chimeric mouse was crossed to a FLPer mouse that ubiquitously expressed Flp-recombinase (Jax Lab # 009086) (2). Flp-recombinase-mediated excision of the *Lac*Z-*Neo* cassette produced progeny with a single conditional *Ric-8A* allele harboring a floxed exon 5. These mice were intercrossed to produce *Ric-8A*^Flox/Flox^ mice (3). **(B)** All mouse genotypes were verified using the indicated genomic DNA PCR genotyping strategies. The following oligonucleotide primers were used:

1: 5’-GTTCCACGGGTGAGGGGTAGGGC-3’

2: 5’-CCAACTGACCTTGGGCAAGAACAT-3’

3: 5’-CCGTGTGGCTGGGGACAGACAC-3’

4: 5’-CTAGCAACATGGCTCCTCAGATCTAATG-3’

PCR primer set 1 and 4 was used to distinguish between *Ric-8A*-Wt, Flox and knockout alleles. PCR was performed using 200 ng of genomic DNA extracted from mouse tail tips using a Qiagen DNAeasy^®^ Blood and Tissue kit with the program: 35 cycles of 94 ^o^C for 1 min, 60 ^o^C for 45 sec, 72 ^o^C for 2 min, followed by a 5 min extension phase at 72 ^o^C. **(C)** Characterization of *Ric-8A*^Flox^ and *Ric-8A*^Knockout^ allele mRNA transcripts. Total RNA was isolated from *Ric-8A*^Flox/Flox^ MEFs that had been treated with or without Cre-lentivirus. The RNA was subjected to reverse transcription / PCR as illustrated in the schematic using following PCR primer sets:

**1. Exon 2-4 primer set:**

Exon1/2 junction forward: 5’-cgttcaaccgggagcattctcagagc-3’

Exon 4 reverse: 5’-GTGGAACTCCTCTGTGCGGTCCCCAG-3’

**2. Exon 4-7 primer set:**

Exon 4 forward: 5’-ccggtacctggggactcttctgcggc-3’

Exon 7 reverse: 5’-CA TCTGTATCCAGGTGTGTCATGAGG-3’

**3. Exon 4-8 primer set:**

Exon 4 forward: 5’-ccggtacctggggactcttctgcggc-3’

Exon 8/9 junction reverse: 5’-CAGGGTTGATGCTGGCCTTGGCTTC-3’

PCR products were resolved on ethidium-bromide 1% Agarose gels and visualized by UV transillumination. The Exon 2-4 primer set amplified a 743 bp product that was common to the Cre treated and untreated MEFs. The Exon 4-7 and Exon 4-8 primer sets amplify products that would span Exon 5 in the WT *Ric-8A* allele, but due to Cre-mediated excision of Exon 5, smaller PCR products were obtained with both primer sets using cDNA obtained from Cre-treated MEFs.

**(D)** The Exon 4-8 primer set RT-PCR products obtained from Cre-treated and untreated MEFs were sequenced. Cre-mediated excision of exon 5 in *Ric-8A*^Flox/Flox^ MEFs resulted in a transcript that had a premature stop codon 3’ of the new junction of exon 4 and exon 6. If a stable 1-276 amino acid Ric-8A truncated protein were produced from this shortened transcript (full length protein is 531 total amino acids), it would lack all ability to act as a GEF and/or fold G protein α subunits as described (4, 5). **(E)** *Ric-8A*^Flox/Flox^*; Rosa-CreER^+/+^* mice were generated by crossing a *Ric-8A^Flox/Flox^* mouse to a R26-Cre-ER^T2^ mouse (Jax Lab # 008463) and backcrossing to achieve the desired double homozygous genotype. The genotype was confirmed by genomic DNA PCR analysis using Cre specific primers (Jax Lab #s IM8545, IM8546 and IM8547) and PCR primer sets 1 and 3 [from **(B)**] to amplify the *Ric-8A* Flox allele. *Ric-8A^Flox/Flox^*, *Ric-8A^Flox/Flox^; Rosa-CreER^+/+^,* and *Ric-8A^Flox/Flox^; Rosa-CreER^+/-^* mice were viable with no obvious defects and generated normal size litters at the expected Mendelian ratios.

**Supplementary Fig. 2.**

Cre-NLS-lentiviral infection of cultured or *Ric-8A*^Flox/Flox^ MEFs (A) resulted in genomic deletion of *Ric-8A* exon 5 and (B) reduced Ric-8A protein abundance and the levels of Gα subunits folded by Ric-8A (Gαq/11, Gαi_1/2_ and Gα13).

**Supplementary Fig. 3.**

Generation of *Ric-8A^Flox/Flox^; Rosa-CreER^+/-^* melanocyte cell lines that stably express *GNAQ*^Q209L^, *GNAQ*^WT^, or GFP and derivation of *GNAQ*^Q209L^ melanoma cell lines from explanted tumors. **(A)** The parental *Ric-8A*^Flox/Flox^; *Rosa-CreER^+/-^* melanocyte cell line was transduced with lentiviruses that expressed *GNAQ*^Q209L^-IRES-GFP, *GNAQ*^WT^-IRES-GFP or -IRES-GFP alone. Stable expression was achieved by cassette selection with hygromycin B and verified by Western blotting for Gαq-Q209L or overproduced Gαq (not shown), and by visualization of uniform GFP fluorescence across the cell populations by microscopy. **(B)** *Ric-8A*^Flox/Flox^; *Rosa-CreER^+/-^*, Tg (*GNAQ*^Q209L^-IRES-GFP) primary melanoma tumors grown in NSG mice were excised and melanoma cell lines were cultured *ex vivo* in standard melanocyte culture medium lacking TPA (one of two tumor explants is shown). The cell line was treated with 4-hydroxy tamoxifen to induce *Ric-8A* knockout, followed by culture medium supplementation with TPA to support growth due to *Ric-8A* knockout-mediated loss of Gαq-Q209L abundance. This *Ric-8A*^KO/KO^; *Rosa-CreER^+/-^*, Tg (*GNAQ*^Q209L^-IRES-GFP) melanoma cell line retained efficient production of GFP fluorescence as determined by microscopy.

**References:**

1. Testa G, Schaft J, van der Hoeven F, Glaser S, Anastassiadis K, Zhang Y*, et al.* A reliable lacZ expression reporter cassette for multipurpose, knockout-first alleles. *Genesis*. 2004;38(**3**):151-158.

2. Farley FW, Soriano P, Steffen LS, Dymecki SM. Widespread recombinase expression using FLPeR (flipper) mice. *Genesis*. 2000;28(**3-4**):106-110.

3. Gabay M, Pinter ME, Wright FA, Chan P, Murphy AJ, Valenzuela DM*, et al.* Ric-8 proteins are molecular chaperones that direct nascent G protein alpha subunit membrane association. *Sci Signal*. 2011;4(**200**):ra79.

4. Oner SS, Maher EM, Gabay M, Tall GG, Blumer JB, Lanier SM. Regulation of the G-protein regulatory-Galphai signaling complex by nonreceptor guanine nucleotide exchange factors. *J Biol Chem*. 2013;288(**5**):3003-3015.

5. Thomas CJ, Briknarova K, Hilmer JK, Movahed N, Bothner B, Sumida JP*, et al.* The nucleotide exchange factor Ric-8A is a chaperone for the conformationally dynamic nucleotide-free state of Galphai1. *PLoS One*. 2011;6(**8**):e23197.
