# Supplementary figures and images for: Ric-8A gene deletion or phorbol ester suppresses tumorigenesis in a mouse model of GNAQQ209L-driven melanoma
Source: Oncogenesis. 2016 Jun 27;5(6):e236–. doi: 10.1038/oncsis.2016.45 (PMC4945744; doi:10.1038/oncsis.2016.45)

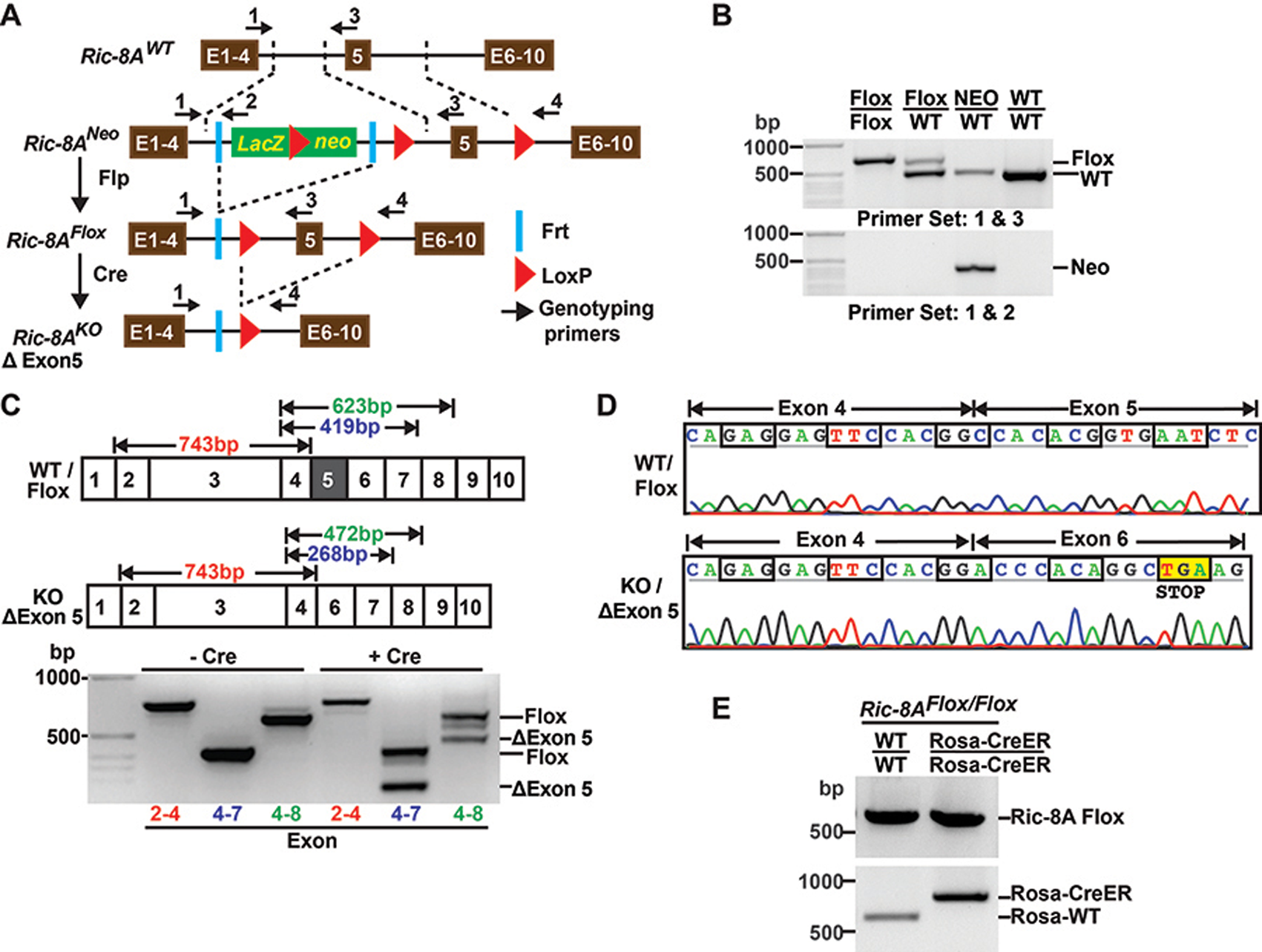

Supplement: Supplementary Figure S1 [file oncsis201645x2.tif]

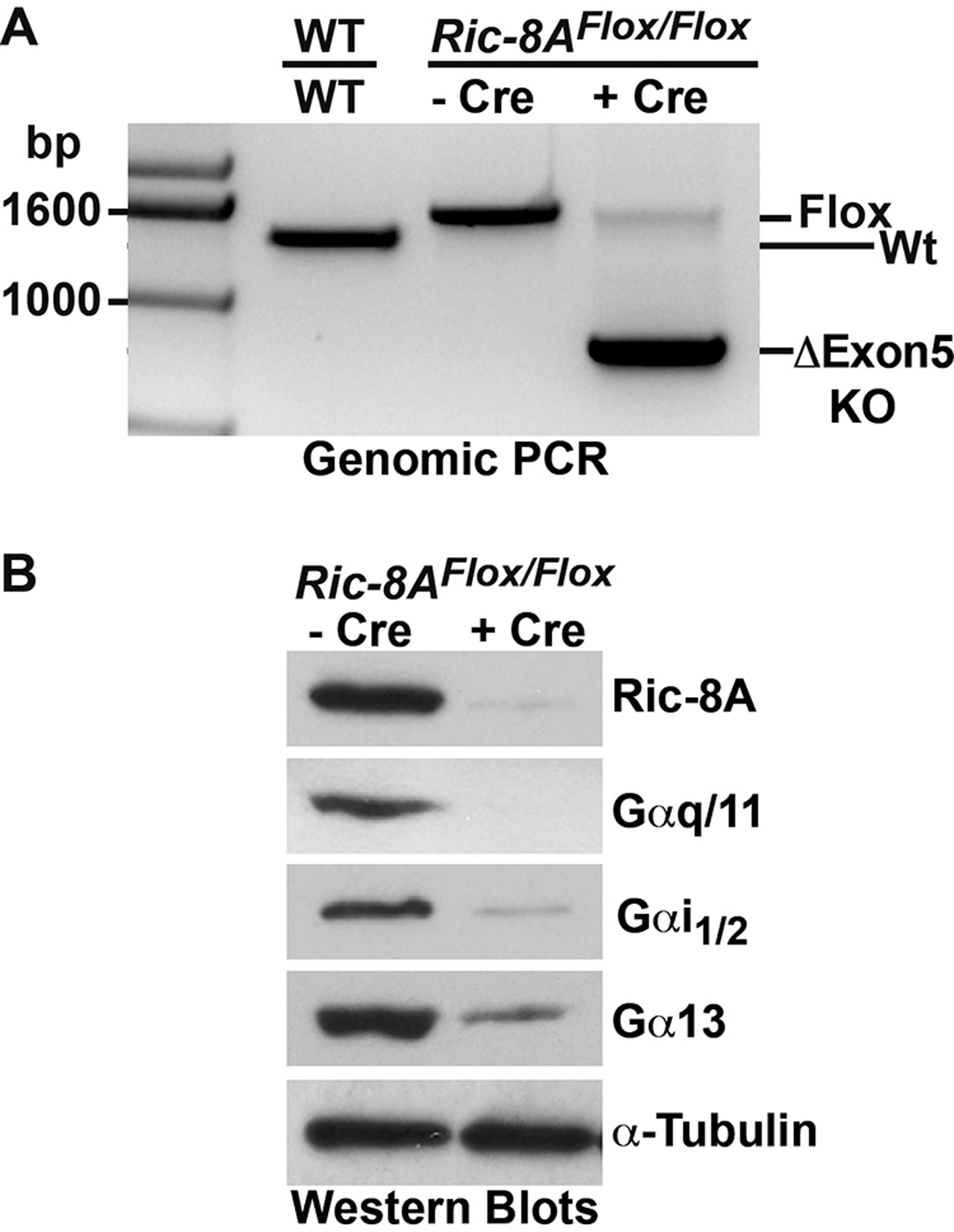

Supplement: Supplementary Figure S2 [file oncsis201645x3.tif]

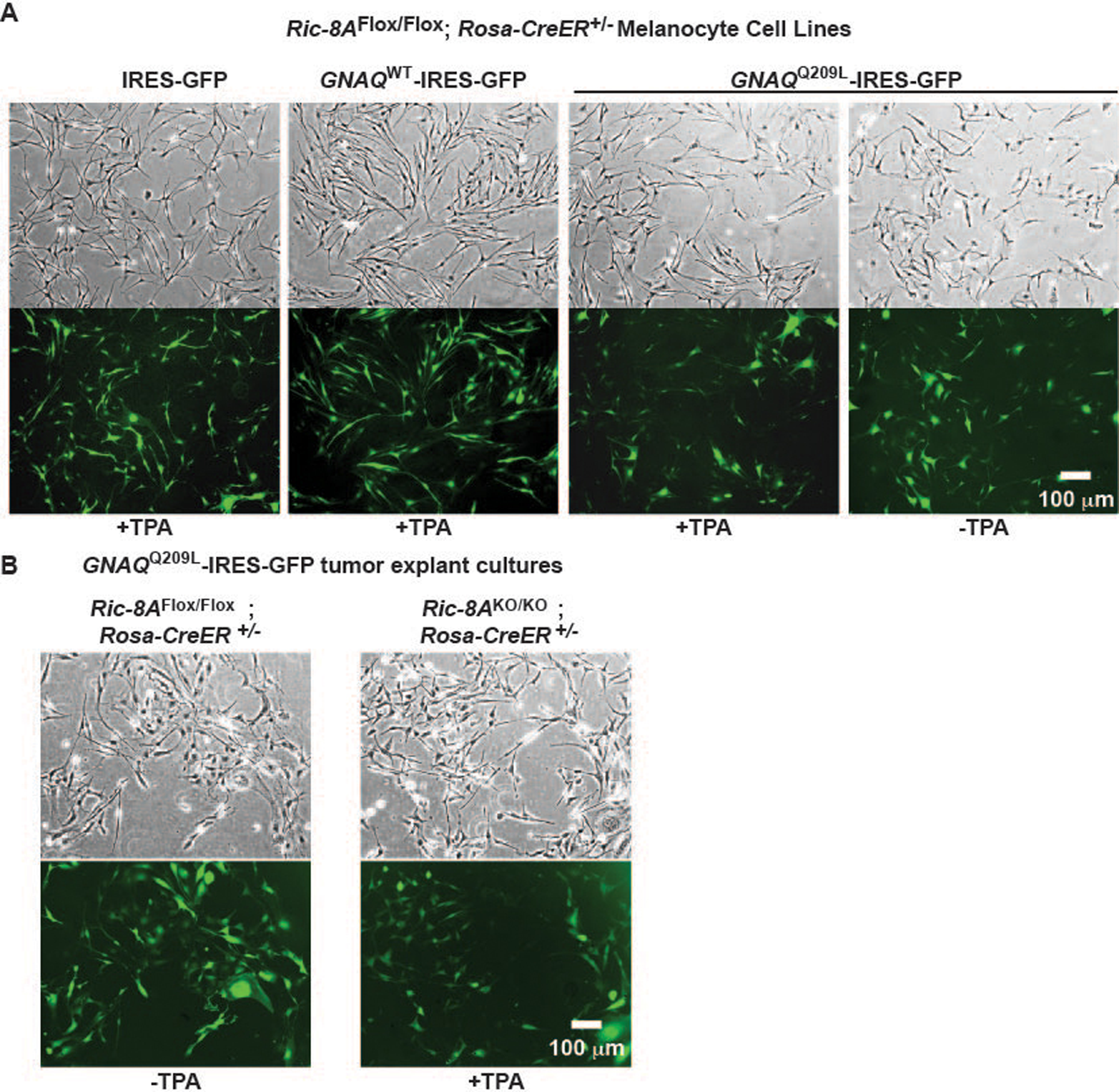

Supplement: Supplementary Figure S3 [file oncsis201645x4.tif]
